# Replication study reveals miR-483-5p as an important target in prevention of cardiometabolic disease

**DOI:** 10.1186/s12872-021-01964-0

**Published:** 2021-04-01

**Authors:** Widet Gallo, Filip Ottosson, Cecilia Kennbäck, Amra Jujic, Jonathan Lou S. Esguerra, Lena Eliasson, Olle Melander

**Affiliations:** 1 Department of Clinical Sciences-Malmö, Hypertension and Cardiovascular Disease, Lund University, 205 02 Malmö, Sweden; 2 Department of Clinical Sciences Malmö, Lund University Diabetes Centre, Lund University Malmö, Malmö, Sweden; 3 Clinical Research Centre, Skane University Hospital, Lund and Malmö, Malmö, Sweden; 4 Department of Emergency and Internal Medicine, Skane University Hospital, Malmö, Sweden; 5 Department of Cardiology, Skane University Hospital, Malmö, Sweden; 6 Department of Clinical Sciences-Malmö, Islet Cell Exocytosis, Lund University, Malmö, Sweden; 7 Department of Clinical Sciences-Malmö, Clinical Research Centre, CRC, Lund University, 91:12, Jan Waldenströmsgata 35, 214 28 Malmö, Sweden

**Keywords:** MiR-483-5p, Cardiometabolic disease, Atherosclerosis, Diabetes mellitus

## Abstract

**Background:**

Alterations in levels of circulating micro-RNAs might reflect within organ signaling or subclinical tissue injury that is linked to risk of diabetes and cardiovascular risk. We previously found that serum levels of miR-483-5p is correlated with cardiometabolic risk factors and incidence of cardiometabolic disease in a case–control sample from the populations-based Malmö Diet and Cancer Study Cardiovascular Cohort (MDC-CC). We here aimed at replicating these findings and to test for association with carotid atherosclerosis.

**Methods:**

We measured miR-483-5p in fasting serum of 1223 healthy subjects from the baseline examination of the population-based, prospective cohort study Malmö Offspring Study (MOS) and correlated miR-483-5p to cardiometabolic risk factors and to incidence of diabetes mellitus and coronary artery disease (CAD) during 3.7 (± 1.3) years of follow-up using logistic regression. In both MOS and MDC-CC we related mir-483-5p to carotid atherosclerosis measured with ultrasound.

**Results:**

In cross-sectional analysis miR-483-5p was correlated with BMI, waist circumference, HDL, and sex. After adjustment for age and sex, the association remained significant for all risk factors except for HDL. Logistic regression analysis showed significant associations between miR-483-5p and new-onset diabetes (OR = 1.94, 95% CI 1.06–3.56, *p* = 0.032) and cardiovascular disease (OR = 1.99, 95% CI 1.06–3.75, *p* = 0.033) during 3.7 (± 1.3) years of follow-up. Furthermore, miR-483-5p was significantly related with maximum intima-media thickness of the carotid bulb in MDC-CC (*p* = 0.001), but not in MOS, whereas it was associated with increasing number of plaques in MOS (*p* = 0.007).

**Conclusion:**

miR-483-5p is related to an unfavorable cardiometabolic risk factor profile and predicts diabetes and CAD, possibly through an effect on atherosclerosis. Our results encourage further studies of possible underlying mechanisms and means of modifying miR-483-5p as a possible interventional target in prevention of cardiometabolic disease.

## Background

Both cardiovascular diseases (CVD) and diabetes are connected to death and large suffering for the society. According to WHO one third of global death in 2016 (17.9 million people) was due to cardiovascular diseases (CVD) [1] and according to IDF about 460 million suffer from diabetes [2]. CVD is commonly caused by atherosclerosis [3] whereas type 2 diabetes (T2D) is characterized by elevated blood glucose raised mainly from defects in insulin secretion and insulin resistance [4]. In T2D and CVD, collectively named cardiometabolic diseases, some of the risk factors are obesity, dyslipidemia, hyperglycemia and hypertension [5]. Diabetes itself is a potent risk factor for CVD. Still, the residual risk is significant. For example, half of patients with established coronary heart disease have only one or no established CVD risk factors at all [6]. Thus, there is a need of identification of novel risk factors. Moreover, diabetes continues to increase globally [7] and even prediabetic stages put individuals at increased risk of CVD [8]. This suggests that one of the most efficient ways to prevent CVD would be to identify novel modifiable risk factors for both T2D and CVD.

MicroRNAs have been shown to be involved in the development of both T2D [9, 10] and CVD [11]. MicroRNAs are endogenous 17–23 nucleotide-long, non-coding RNAs that mediate post-transcriptional gene silencing by binding to the 3′untranslated regions (UTRs) on target mRNA, resulting either in translational inhibition or mRNA degradation [12]. Due to their function as important regulators of gene expression within the cytoplasm of various cells and their ability to be released into the circulation, microRNAs might be attractive tools in therapeutics of disease as discussed in [13]. microRNAs are stable in body fluids and are supposed to be protected from degradation through binding to exosomes, RNA-binding proteins or lipoproteins [9, 14]. The release of microRNAs from different cells is suggested to be cell-specific and could therefore participate in signaling between organs, i.e. acting in a similar way as hormones [14]. We recently made the novel discovery that circulating levels of miR-483-5p correlate with several risk factors of diabetes and CVD and that expression of this microRNA predicts development of diabetes and CVD later in life in the Malmö Diet and Cancer study Cardiovascular-Cohort (MDC-CC). Interestingly, the relationship between miR-483-5p expression and risk of both diseases remained significant after adjustment for traditional risk factors, although the association with diabetes incidence was attenuated [15].

In the present study, we aim to replicate the aforementioned associations between mir-483-5p and cardiometabolic disease and its risk factors. Moreover, we hypothesize that the association between circulating levels of miR-483-5p and incident CVD is driven by atherosclerosis. Therefore, we also investigate whether serum miR-483-5p expression is associated with measures of carotid atherosclerosis.

## Methods

### Subjects and endpoints

#### Malmö offspring study (MOS)

MOS is a family-based cohort study that was initiated in 2013. The participants of MOS consists of the children and grandchildren of a bigger population-based cohort, the Malmö Diet and Cancer-cardiovascular cohort (MDC-CC). Individuals recruited were ≥ 18 years old, living in Malmö (southern Sweden). Anthropometrics measurements (height, weight, waist, hip circumference) were carried out and fasting venous blood was drawn. For this paper, 1223 serum-samples were stored at – 80 ℃ until analysis.

#### Malmö diet and cancer-cardiovascular cohort (MDC-CC)

The Malmö Diet and Cancer study (MDC) is a population-based prospective cohort from Sweden, where participants were examined between 1991 and 1996 (n = 28 449). For further examination and with the primary aim of study the epidemiology of carotid artery diseases, (n = 6103) of all participants was randomly selected between 1991 and 1994. This sub-cohort is referred as The Malmö Diet and Cancer-Cardiovascular Cohort. We selected 553 individuals from the MDC-CC for analysis of miR-483-5p, as described in detail previously [15].

### Samples

Serum samples of 1223 subjects from the baseline examination of the population-based, prospective cohort study Malmö Offspring Study (MOS) were used in this study. The study was approved by Regional Ethics Committee in Lund University, Sweden. Both written and oral informed consent was obtained from all patients.

### MiRNA-measurements

Briefly, 250 µl human serum samples were placed into a plate followed by centrifuging for 5 min at 1000 g at 4 °C. Total RNA was extracted from 200 μl serum using the miRNeasy 96 Total RNA isolation kit (Qiagen) using a modified protocol (see or previous study [15]).

For cDNA synthesis, we performed a modified protocol starting with the poly(A) tailing followed by adaptor ligation, reverse transcriptase reaction and miR-Amp reaction according to manufacturer’s recommendations for TaqMan Advanced NA Assays (Cat# A25576). For the cDNA template, the dilution was changed to 1:5.

For performing qPCR, we used custom plated 384-well TaqMan plates (Thermofisher Scientific) with eight unique miRNA assays per plate. The assays used were as follows: hsa-miR-483-5p (Assay ID: 478432_mir), hsa-miR-222-3p (Assay ID: 477982_mir), hsa-miR-17-5p (Assay ID: 478447_mir), hsa-miR-320a (Assay ID: 478594_mir), hsa-miR-223-3p (Assay ID: 477983_mir), hsa-miR-338-5p (Assay ID: 478038_mir), hsa-miR-126-3p (Assay ID: 477887_mir), hsa-let7-5p (Assay ID: 478579_mir).

Real-time PCR was performed according to manufacturer’s recommendation on ViiA 7 Real-Time PCR System (Thermofisher Scientific).

Criteria for inclusion/exclusion of samples before normalization and analysis.

For hsa-miR-17-5p assay, only samples between C_t_-values 20.1–28.3 were included.

For the remaining seven assays, only samples with C_t_-values ≥ 20 were included. All undetermined assays were coded as 40; that resulted in 1223 samples for the data analysis.

All C_t_ -values from selected samples were log-transformed and normalized against log-transformed hsa-miR-17-5p. The hsa-miR-17-5p was used because of its ‘stability and low variation between samples, which also was identified by Gidlöf et.al [16] as a stable normalizer.

### Statistics

Because of non-normal distributions, the expression level of miR-483-5p was log-transformed prior to statistical analysis, and then z-transformed. Pearson’s correlation tests were performed between cardiometabolic risk factors and miR-483-5p in 1223 subjects. Further partial Pearson’s correlation tests, adjusted for age and sex, were performed between miR-483-5p and HDL cholesterol, triglycerides, BMI, waist circumference and fasting glucose. Age and sex adjusted logistic regression was used to calculate odds ratios (OR) per one standard deviation increment of standardized scaled miR-483-5p expression levels for incident MACE and incident diabetes, whereas linear regression was used to calculate the correlation between miR-483-5p and bulb-IMT both in MOS and MDC-CC and between miR-483-5p and the number of plaques in MOS. The number of plaques was defined as zero for absence of plaques, one for one plaque and two for two or more plaques. All statistical analyses were performed in IBM SPSS Statistics for Windows, version 26.0 (Armonkulb, New York, United States).

### Ultrasound of the carotid arteries

#### Malmö diet and cancer: cardiovascular cohort

Ultrasound examinations were performed on the right carotid artery using an Acuson Seqouia (Acuson, Mountain View, Calif.) with a 7 MHz transducer. Images were obtained in a pre-defined window (3 cm of the distal part of common carotid artery, the bifurcation area and 1 cm of proximal internal and external carotid artery respectively) and captured at end-diastole. Measurements of IMT-bulb were carried out off-line, using the Artery Measurement System (AMS), and a mean of three measurements was calculated. A complete description of the procedure is given elsewhere [17].

### Malmö offspring study

Ultrasound examinations were carried out using Logiq E9 (GE Healthcare). Images of the left and right common carotid artery and the bifurcation were obtained in a predefined window and captured at end-diastole. Measurements of IMT-bulb were performed out off-line using the AMS program. A plaque was defined as a focal thickening of the intima layer with a height > 1.2 mm. All plaques visible in the bifurcation, and in the common, internal, and external were reported.

### Clinical measurements and endpoint definitions of study cohorts

Clinical examinations were performed for all participants in both MDC and MOS. Body-mass Index (BMI) was defined as the weight divided by the squared height (kg/m^2^). Systolic and diastolic blood pressure (mmHg) were measured using the mercury-column sphygmomanometer. Analyses of fasting glucose, High-density Lipoprotein (HDL) and triglycerides were carried out using routine standards methods at the Department of Clinical Chemistry, Malmö. Low-density Lipoprotein (LDL) was calculated using the Friedwald equation. Insulin levels measurements were performed using Mercodia Insulin ELISA (Mercodia, Uppsala, Sweden).

Type 2 Diabetes (T2D) was defined as a fasting plasma glucose > 7 mmol/L or a history of physician diagnosis of T2D or being on antidiabetic medication or having been registered in local or Swedish diabetes registries [18] and Coronary Artery Disease (CAD) as myocardial infarction (fatal and non-fatal), death due to ischemic heart diseases or coronary revascularization as described previously [19, 20].

## Results

Participants in MOS, in whom miR-483-5p was measured, consisted of 1223 individuals with the mean age of 42 years, 52% were women. In MDC-CC, the corresponding number was 553 individuals with a mean age of 59 years and 51% women (Table 1). As expected, the population-based MOS had more favorable values of several risk factors than the MDC-CC participants had. This is in part a result of the fact that MOS were younger at their baseline examination and partly that the sample from MDC-CC consisted of incident CVD cases, incident diabetes cases and control subjects [15, 21].

**Table 1:** Baseline clinical characteristics of subjects in MOS and MDC-CC

	MOS		MDC-CC	
	N	Mean (SD)	N	Mean (SD)
Age (years)	1221	41.62 ± 13.6	553	59.16 ± 5.8
Women (%)	1221	51.8	553	51.2
BMI (kg/m^2^)	1221	26.04 ± 4.68	553	27.06 ± 4.56
Waist circumference (cm)	1221	90 ± 14	553	89 ± 14
Fasting glucose (mmol/L)	1219	5.48 ± 0.96	533	5.37 ± 1.26
LDL cholesterol (mmol/L)	1217	3.21 ± 1.02	553	4.27 ± 0.92
HDL cholesterol (mmol/L)	1218	1.6 ± 0.48	553	1.29 ± 0.33
Triglycerides (mmol/L)	1208	1.17 ± 0.70	553	1.47 ± 0.70
Systolic blood pressure (mmHG)	1165	120.62 ± 17.33	553	147.69 ± 19.97
Diastolic blood pressure (mmHG)	1165	83.33 ± 10.71	553	89.72 ± 9.57

In cross-sectional correlation analyses of 1223 subjects from MOS, miR-483-5p was positively correlated to body mass index (BMI), waist circumference, triglyceride concentrations, and male sex and negatively correlated to High Density Lipoprotein -cholesterol (HDL-C). However, no significant correlation with fasting glucose or age was observed (Table 2). The correlations with cardiometabolic risk factors remained significant after adjustment for age and sex, except for HDL (Table 2).

**Table 2:** Correlation between miR-483-5p and diabetes risk factors in MOS

	Age	Sex	HDL	Triglycerides	BMI	Waist	Glucose
N	1221	1221	1218	1208	1221	1221	1219
Pearson correlation	0.02	− .100^**^	− .085^**^	.100^**^	.099^**^	.119^**^	0.023
*p* value	0.40	0.0004	**0.003**	** < 0.001**	**0.001**	** < 0.001**	0.432
Partial Pearson correlation			− .050	.076	0.078	0.082	0.006
*p* value			0.083	**0.008**	**0.007**	**0.004**	0.841

Pearson Correlation: miR-483-5p correlated positively with Triglycerides, Body Mass Index (BMI), Waist and negatively with High Density Lipoprotein (HDL)

Partial Correlation: miR-483-5p correlated positively with Triglycerides, Body Mass Index (BMI) and Waist

Bold indicates *P*-value < 0.01

**Correlation is significant at the 0.01 level (2-tailed). * Correlation is significant at the 0.05 level (2-tailed)

Next, we replicated in MOS, our previous finding in MDC-CC that the level of miR-483-5p is associated with incidence of diabetes. Among subjects in MOS free from diabetes at the time of the baseline exam (N = 1177), 12 individuals developed diabetes during 3.7 (± 1.3) years of follow-up. Baseline level of miR-483-5p was significantly associated with incident diabetes in an age and sex adjusted logistic regression model (odds ratio (OR) per one standard deviation (SD) increment of miR-483-5p = 1.94, 95% confidence interval (CI95%) = 1.06–3.56, *p* = 0.032).

Given our previous finding on an association between miR-483-5p serum levels and incident cardiovascular disease in the MDC [15], we subsequently related levels of miR-483-5p to the maximum intima-media thickness in the bulb (IMT-bulb) region in the MDC-CC. In concordance with our previously reported association with incident cardiovascular disease, we found a significant association between miR-483-5p and IMT-bulb (Table 3). Furthermore, we cross-sectionally evaluated the relationship between miR-483-5p and carotid atherosclerosis in MOS, where both thickness of the IMT-bulb (N = 485) and number of carotid plaques (N = 699) were measured in a subset of the participants (N = 1223). Whereas there was no association between levels of miR-483-5p and thickness of the IMT-bulb in MOS, serum levels of miR-483-5p was significantly positively related to number of carotid plaques (Table 3). Finally, among subjects free from coronary artery disease (CAD) in MOS (n = 1208), 14 incident cases of CAD occurred during 3.7 (± 1.3) years of follow-up. Baseline level of miR-483-5p was significantly associated with incident CAD in logistic regression models adjusted for age and sex (per SD increment of miR-483-5p: OR = 1.99, CI 95% = 1.06–3.75, *p* = 0.033).

**Table 3:** Association between miR-483-5p and IMT-bulb and the number of Plaque respectively

	IMT-bulb (mm)				No.of plaques^b^	
	MDC-CC (N = 401)		MOS (N = 485)		MOS (N = 699)	
	Beta	*p* value	Beta	*p* value	Beta	*p* value
miR-483-5p	0.196 (0.08–0.22)^a^	** < 0.001**	0.023 (-0.02–0.04)	0.56	0.09 (0.02–0.15)^a^	**0.007**

Bold indicates *P*-value < 0.01

Age and sex adjusted

^a^Beta-coefficients are expressed per one SD increment of log-transformed miR-483-5p

^b^Indicates the number of plaques: 0 = absence of plaques, 1 = 1 plaque and 2 = > 1 plaques

## Discussion

Our data confirm our previous findings and suggests serum level of miR-483-5p as a strong joint candidate for prediction of new-onset diabetes and CVD. In the present study, we replicated the previously shown cross-sectional associations between serum levels of miR-483-5p and BMI, waist and triglycerides and the prospective associations with new-onset diabetes and cardiovascular disease. Moreover, associations between levels of miR-483-5p and presence of carotid plaques and IMT indicates that the association between miR-483-5p and CVD may be driven by atherosclerosis.

In our previous study, levels of miR-483-5p was for the first time shown to be related to cardiometabolic risk factors and incidence of cardiometabolic disease [15]. Here, we replicate the positive correlations with BMI, waist, and triglyceride levels, whereas the correlation with HDL was borderline significant after adjustment for age and sex.

Despite a low number of incident cases of diabetes, we were able to replicate the previously shown association between miR-483-5p and incident diabetes. It is unknown if elevated level of miR-483-5p is cause or consequence of a dysmetabolic state, and regardless of which one of these two possibilities is true, the underlying mechanisms remain to be unraveled. miR-483-5p was identified in human fetal liver clones in 2005 [22] and is an intronic miRNA located within the gene encoding *IGF-II (*Insulin-like growth factor-2) on chromosome 11p15.5 in humans. miR-483-5p is co-expressed with IGF-II gene and several studies have shown an association between IGF-II and obesity [23–25]. Thus, one can speculate that miR-483-5p regulates IGF-II, which in turn contributes to the dysmetabolic profile that is observed in subjects with high miR-483-5p levels. On the other hand, we measured miR-483-5p in the circulation rather than in the liver. Elevated levels of circulating miR-483-5p can either reflect increased organ leakage of miR-483-5p, for example due to subclinical Non-Alcoholic Fatty Liver Disease (NAFLD) [26] and mild hepatitis, which in turn is associated with high cardiometabolic risk. Another possibility is that miR-483-5p participates in endocrine-like signaling between organs, where increased production of miR-483-5p in e.g. the liver, might be a primary event, acting on other organs such as fat tissue, promoting dysmetabolic processes leading to increased cardiometabolic risk. Importantly, functional studies in vitro and in vivo are needed to shed more light on both cause-consequence relationships and mechanisms underlying the relationship between miR-483-5p and cardiometabolic risk.

As for the previous association between miR-483-5p and CVD, we provide some evidence of replication in the significant associations between miR-483-5p and CAD, although also here limited by a low number of incident cases. As the unfavorable levels of cardiometabolic risk factors, which were associated with high miR-483-5p levels at baseline in both studies, may affect risk of clinical cardiovascular events in numerous ways, e.g. through promotion of atherosclerosis, hypercoagulation, hemodynamic factors and plaque instability, we were interested in direct association with atherosclerosis. Our finding of association between levels of miR-483-5p and measures of carotid atherosclerosis support a direct or indirect effect of miR-483-5p on atherosclerosis. It should, however, be pointed out that the associations with carotid atherosclerosis were not fully concordant between the two studies. One possibility that could explain lack of association with IMT-bulb thickness in MOS, is the younger age of this population compared to MDC-CC. IMT-bulb might be driven by relatively large plaques in the bifurcation region, which would be less common in a younger population. On the other hand, the number of plaque measure in MOS takes into account presence of several small plaques, which individually would only marginally affect the thickness of the IMT-bulb. This may explain the significant association with the number of plaques in MOS. Unfortunately, we were not able to measure the association between levels of miR-483-5p and the number of plaques in MDC-CC due to the different study design in this cohort.

Both carotid plaques [27] and IMT-bulb have been used as markers for atherosclerosis, for prediction of myocardial infarction and stroke, where IMT-bulb has been shown to be more related to incident atherosclerosis [28–30]. Thus, we hypothesize that atherosclerosis is the link that elucidates the relationship between miR-483-5p and CAD.

There are some limitations to this study. First, the low number of incident diabetes (N = 12) and CAD (N = 14) subjects, a consequence of participants in MOS being relatively young and healthy, and that the follow-up time is short. Moreover, since the participants in MOS are young and healthy compared to MDC, it might not be an ideal cohort to study IMT. Mor studies with longer follow-up are needed to confirm our findings. However, despite the low number of incident cases, we do observe significant association between levels of miR-483-5p and incident diabetes and CAD. Finally, although miR-483-5p is a promising biomarker, it is necessary with functional analysis and determining of the target tissue to discover novel pathways that may help us understand how miR-483-5p is released into the circulation.

The strengths of our study are firstly, the quantity of subjects included i.e., with 1223 individuals this is the largest population-based cohort used to study miR-483-5p. Secondly, the success in replicating previous findings by which we have been able to confirm that miR-483-5p associates with cardiometabolic risk factors and incidence of diabetes and CAD.

## Conclusion

From this study is that miR-483-5p is a very attractive candidate and a non-invasive biomarker for prediction of cardiovascular diseases and diabetes, and therefore it deserves further investigations.

## Data Availability

Supporting data will be made available upon request to the corresponding authors.

## Abbreviations

BMI: Body Mass Index
CAD: Cardio Artery Disease
CVD: Cardiovascular Disease
HDL: High Density Lipoprotein
IGF-II: Insulin-like Growth Factor-2
IMT: Intima-Media Thickness
LDL: Low Density Lipoprotein
MDC: Malmö Diet and Cancer Study
MDC-CC: Malmö Diet and Cancer Study-Cardiovascular Cohort
MOS: Malmö Offspring Study
NAFLD: Nonalcoholic Fatty Liver Disease
OR: Odds Ratio
SD: Standard Deviation
T2D: Type 2 diabetes
